# HOX gene complement and expression in the planarian *Schmidtea mediterranea*

**DOI:** 10.1186/s13227-016-0044-8

**Published:** 2016-03-30

**Authors:** Ko W. Currie, David D. R. Brown, Shujun Zhu, ChangJiang Xu, Veronique Voisin, Gary D. Bader, Bret J. Pearson

**Affiliations:** Program in Developmental and Stem Cell Biology, Hospital for Sick Children, Toronto, ON M5G10A4 Canada; Department of Molecular Genetics, University of Toronto, Toronto, ON M5G10A4 Canada; Ontario Institute for Cancer Research, Toronto, ON M5G10A4 Canada; Donnelly Centre for Cellular and Biomolecular Research, Toronto, ON M5G10A4 Canada

**Keywords:** In situ hybridization, Body plan, HOX, Homeotic, Lophotrochozoan, Flatworm, Planarians, *Schmidtea mediterranea*

## Abstract

**Background:**

Freshwater planarians are well known for their regenerative abilities. Less well known is how planarians maintain spatial patterning in long-lived adult animals or how they re-pattern tissues during regeneration. HOX genes are good candidates to regulate planarian spatial patterning, yet the full complement or genomic clustering of planarian HOX genes has not yet been described, primarily because only a few have been detectable by in situ hybridization, and none have given morphological phenotypes when knocked down by RNAi.

**Results:**

Because the planarian *Schmidteamediterranea* (*S. mediterranea*) is unsegmented, appendage less, and morphologically simple, it has been proposed that it may have a simplified HOX gene complement. Here, we argue against this hypothesis and show that *S. mediterranea* has a total of 13 HOX genes, which represent homologs to all major axial categories, and can be detected by whole-mount in situ hybridization using a highly sensitive method. In addition, we show that planarian HOX genes do not cluster in the genome, yet 5/13 have retained aspects of axially restricted expression. Finally, we confirm HOX gene axial expression by RNA deep-sequencing 6 anterior–posterior “zones” of the animal, which we provide as a dataset to the community to discover other axially restricted transcripts.

**Conclusions:**

Freshwater planarians have an unappreciated HOX gene complexity, with all major axial categories represented. However, we conclude based on adult expression patterns that planarians have a derived body plan and their asexual lifestyle may have allowed for large changes in HOX expression from the last common ancestor between arthropods, flatworms, and vertebrates. Using our in situ method and axial zone RNAseq data, it should be possible to further understand the pathways that pattern the anterior–posterior axis of adult planarians.

**Electronic supplementary material:**

The online version of this article (doi:10.1186/s13227-016-0044-8) contains supplementary material, which is available to authorized users.

## Background

HOX genes are well-known, conserved regulators of axial body patterning during embryogenesis in most studied animals [1–6]. When HOX genes are mutated, homeotic transformations result where one body region or segment will take on that of another, such as transforming fly antennae into legs [7]. In addition, HOX genes tend to exist in clusters in the genome in the exact order on the chromosome in which they are spatially expressed along the anterior–posterior (A–P) axis—a mechanism termed “colinearity” [8, 9]. A typical number of HOX genes in a cluster are between 9 (*Drosophila*) and 14 (amphioxus) [10, 11]. It is generally thought that large changes in body plans between species can be explained by developmental changes in HOX gene regulation or loss of HOX genes altogether [12–15].

As more species have their genomes sequenced, several aspects of the HOX gene dogma are being challenged [10]. It is clear that HOX genes need not be in clusters for colinear (“axially restricted” if no longer in a cluster) expression as has been seen for the urochordates *Oikopleura* and *Ciona* [10]. Furthermore, depending on the organism, HOX genes can be lost (parasitic flatworms: 7 HOX [16–18]; *C. elegans*: 6 HOX [19]), duplicated (vertebrates 39–48 HOX in 4–7 clusters [2, 20–23]), and even retained but function in cells other than axial patterning, typically neural fate specification (*C. elegans*, vertebrates) [22, 23]. Therefore, only studies of HOX genes in a wide variety of organisms can give us a complete picture of HOX gene changes during body plan evolution. Because vertebrates, arthropods, and annelids share the characters of body segmentation and colinear HOX gene expression, it is parsimonious that their common ancestor also had these features. However, many organisms, such as freshwater planarians (triclad Platyhelminthes), many mollusks, *C. elegans* (nematodes), or acoel flatworms (deuterostomes [24]), have neither obvious morphological segmentation nor appendages. This observation raises the question as to whether “simple” body plans equals reduced HOX complements?

Planarians are free-living, freshwater flatworms (triclads) and members of the phylum Platyhelminthes and the super-phylum Spiralia. While planarians have classically been studied for their regenerative abilities [25], they are becoming a molecular model for adult stem cell biology and are beginning to lend an evolutionary perspective on conserved developmental pathways due to their Spiralian phylogenetic position and ability to test gene function by RNA interference (RNAi) [26, 27]. Planarians pose additional questions regarding HOX gene evolution and function. First, asexual *Schmidtea mediterranea* (*S. mediterranea*) are constitutive adults and are no longer capable of embryogenesis [28, 29]. What is the HOX complement in *S. mediterranea*, and have they lost HOX genes coinciding with the loss of embryogenesis? Second, planarians do not have a through-gut and instead have only a single body opening, located centrally and ventrally, which functions as both the mouth and anus. Have planarians changed the location of this opening by altering HOX gene expression or have they retained “normal” axial restriction of HOX gene expression? If the opening is a true mouth, are anterior HOX genes expressed there or are they retained at the anterior where the brain and eyes are located? Conversely, if the opening is a true anus, are posterior HOX genes and/or typical hindgut genes expressed in this location (e.g., *Cdx*, *orthopedia*) [30, 31]? Finally, whereas most species use HOX genes to specify limb or segment identity, planarians have neither limbs nor obvious segments. Have planarians lost HOX genes due to seeming lack of A–P complexity or have they been retained for other reasons such as cell-type specification?

The current study is not the first to examine HOX gene evolution in flatworms, nor the first to pose the above questions. To date, 10 HOX genes have been cloned across 6 species of planarians [3, 18, 31–37]. Several of these studies have shown whole-mount in situ hybridization (WISH) patterns of a posterior gradient of posterior HOX gene homologs in planarians [33, 38, 39]. Other studies have examined HOX gene expression when planarians were forced to regenerate in various ways, and some HOX gene re-expression was observed to occur specifically during regeneration [32–34]. However, no phenotype has been functionally ascribed to planarian HOX genes, nor has the HOX complement of any species of planarians been fully described. Additionally, it has been shown that other signaling pathways, such as WNT/β-catenin, mediate large body region decisions during regeneration [27, 40]. Thus, HOX genes have received little attention since the original incomplete descriptions of several genes across multiple species.

In order to begin to address the many unknowns regarding HOX genes in planarians, here we first describe the entire complement of HOX genes in *S. mediterranea* using genomic and transcriptomic searches with phylogenetic analyses. We show that *S. mediterranea* has a total of 13 HOX genes which represent all major A–P categories. We further show that while the HOX genes are not organized into a genomic cluster, *S. mediterranea* has retained several aspects of canonical axially restricted gene expression. In order to detect the spatial expression of *S. mediterranea* HOX genes, we developed a very sensitive whole-mount in situ hybridization (WISH) technique as well as performed RNA deep sequencing (RNAseq) of 6 A–P zones of the animal to confirm HOX gene WISH detection and to provide this as a resource for future gene discovery. Interestingly, we find that 5 HOX genes have retained axially restricted expression, 2 genes have radial body-edge expression with no axial restriction, and 6 genes have tissue-specific expression. Finally, we show that a posterior HOX gene is expressed in the body opening, and an *orthopedia* homolog is expressed in the pharynx, together suggesting that this body opening is the anus/hindgut and that the mouth has been lost. In total, our results support an unappreciated HOX complexity in planarians, which can now be functionally investigated for roles in spatial patterning during planarian embryogenesis, adult homeostasis, and injury regeneration.

## Methods

### Strain and RNAi

The asexual strain of *Schmidtea mediterranea* CIW4 was used for all experiments [41]. Experimental animals for in situ hybridization and RNAi were size-matched and 4–5 mm in length. All HOX genes were subjected to RNAi feeding every 3 days for >10 feedings as previously described [42]. Half of the animals were amputated into thirds, and the other half were observed for homeostasis defects. In no single-gene RNAi were any defects observed in either regeneration, homeostasis, patterning, or behavior.

### Identification of HOX genes in flatworms

Using the *S. mediterranea* genome database 2.0 (smedgd.stowers.org [43]) and multiple published transcriptomes [42, 44–46], we performed reciprocal TBLASTN with both *Drosophila*, mouse, and polychaete HOX protein sequences. The top 100 homeodomain-containing BLAST hits were tested where top planarian hits were assembled into an open reading frame (ORF) and blasted back to mouse, fly, or NR protein databases at NCBI (http://blast.ncbi.nlm.nih.gov/). If a HOX gene was a top reciprocal hit to mouse and fly, then the *S. mediterranea* sequence was considered to be a HOX gene. There were 13 genes that passed the reciprocal test. All were cloned out of adult planarian cDNA by 3ʹ RACE, and then full length was cloned by 5ʹ RACE. The 3ʹ and 5ʹ RACE products were combined by template-switching PCR. All genes could be cloned from adult tissue, denoting expression in adults.

### Alignment, phylogenetic tree construction, and gene naming

Protein sequences were obtained from the NCBI Entrez protein database as well as individual organism genome sequencing project Web sites and the Homeobox Database (http://homeodb.zoo.ox.ac.uk) [47]. All protein sequences or GenBank accession numbers are in Additional file 1: Table S1. www.macgenome.org was used for all *Macrostomum lignano* genomic and transcriptomic sequences. The program Geneious (www.geneious.com) was used with the MAFFT [48] alignment plugin, and two tree building plugins for Geneious were used for independent analyses. Both maximum likelihood (PHYML) and Bayesian (MrBayes) analyses were performed with WAG amino acid substitution models. PHYML was run with Chi2 branch support statistics with estimated gamma distribution and estimated proportion of invariable sites and set to optimize topology and branch lengths. MrBayes was run with 1.1 million chain length, 4 independent chains, subsample frequency of 1000, invgamma rate variation, and burnin of 25,000. Consensus trees were saved through Geneious as .jpgs, which were then edited to produce final figures in Adobe Photoshop. In 2 cases where a HOX gene could not be specifically assigned to a group (*Smed*-*Hox3b* and *Smed*-*Hox4b*; see “Discussion” section), both were named based on the top reciprocal blast hit homology to the NR database at NCBI as well as the top hits from the Homeobox Database (http://homeodb.zoo.ox.ac.uk) [47].

### Sensitive whole-mount double fluorescent in situ hybridization (dFISH) using FastBlue

FastBlue is an alkaline phosphatase (AP) chromogenic substrate that also fluoresces in far red without blocking wavelengths of standard fluorescent molecules such as FITC, DAPI, or Cy3, and has recently been applied to fluorescent ISH (FISH) in zebrafish [49]. A detailed protocol can be found in Additional file 2 and was a merge of [49–51] with considerable other optimizations. Importantly, we observed that AP developments using FastBlue can be detected fluorescently by confocal microscopy, even when a pattern cannot be seen colorimetrically using either NBT/BCIP or FastBlue (see Additional file 2: Fig. S4). The sensitivity of our protocol, combined with the ability to use a peroxidase-conjugated antibody for another riboprobe and detect both on the same day, leads to minimal chance of false positive overlap as well as shortens the typical dFISH protocol by 1 day, all while achieving much higher sensitivity than has been previously possible. This was a necessary advancement in order to detect the HOX genes.

### RNAseq and bioinformatic identification of zone-specific transcripts

Five adult, starved, 8 mm, asexual *S. mediterranea* animals from the CIW4 strain were immobilized on Peltier plates and carefully amputated into 6 fragments as illustrated in Fig. 2a, and collected directly into Trizol reagent. This process was triplicated, and each RNA sample for each zone was processed independently. Triplicated samples for the same zone were then mixed in equal amounts and submitted for RNAseq. Each zone was sequenced to a depth of >52 million single-end reads on an Illumina HiSeq2500 with v4 chemistry, and libraries were made using an Illumina TruSeq kit using the manufacturer’s protocol. RNAseq data were aligned using bowtie2, with no trimming, to the *S. mediterranea* asexual transcriptome deposited in NCBI under Bioproject PRJNA215411 [46]. Raw sequencing data were uploaded to NCBI GEO under accession number GSE78937.

Specific zones in which transcripts were highly expressed were determined by the edgeR exact test using RNAseq raw counts of the zones 1–6 [52, 53]. The procedure includes data filtering, normalization, differential expression tests, and scoring. There were total 82,827 transcripts detected for the zones 1–6. Transcripts that had <0.5 count per million (CPM) in each zone were filtered out. The CPM cutoff was chosen such that the quantile normalized CPMs have an identical distribution for each zone after filtering the transcripts of extremely low CPM. The filtered transcripts, which had extremely low CPM, could not be found to be specific to any zone. Finally, 24,578 transcripts were kept. The RNAseq raw counts of the zones 1–6 were normalized using the edgeR Trimmed Mean of M-values (TMM) method [52]. The normalization factors are calculated using the TMM method as follows:

$$\begin{aligned} {\text{Effective}}\;{\text{library}}\;{\text{size}}\; & = \;{\text{library}}\;{\text{size}}\;*\;{\text{normalization}}\;{\text{factor}} \\ {\text{Normalized}}\;{\text{CPM}}\; & = \;1{\text{e}}^{6} *\;{\text{counts/effective}}\;{\text{library}}\;{\text{size}} \\ \end{aligned}$$

Box plot, smoothing density plot, hierarchical clustering, and multidimensional scaling (MDS) were used to show the zone relations after normalizing, see Additional file 2: Fig. S1. Using the edgeR exact test, we compared one zone to the average over other five zones to determine whether a transcript was specific to a zone. If the differential expression test for a transcript was significant and the log fold change (logFC) > 0, the transcript was considered to be specific to that zone. A transcript may be specific to more than one zone, in which case the zones will be ordered by scores of the zones, and the zone of higher expression listed first. For example, a transcript with the score “2.1” would mean that it has significant specificity to both zones 1 and 2, with higher expression in zone 2. Zone membership and normalized CPM for all transcripts are in Additional file 3: Table S2 and shown visually in the Additional file 2: Fig. S2 heatmap.

## Results

### *S. mediterranea* has a total of 13, unclustered HOX genes

No individual study of planarian HOX genes has described more than 7, which has begged the question as to how many HOX genes were in the ancestral triclad or Platyhelminth. The ~800 MegaBase *S. mediterranea* genome has been sequenced to >11x coverage and assembled into genomic contigs [43, 54, 55]. We first identified potential HOX genes by reciprocal TBLASTN of every assembled genomic contig containing a homeodomain using HOX sequences from mouse, fly and polychaetes. In addition, we searched 4 published *S. mediterranea* transcriptomes [42, 44–46]. A total of 13 predicted genes had HOX genes as their top, reciprocal BLAST hit. All predicted HOX genes were then cloned by 3ʹ and 5ʹ RACE from total cDNA for verification that they are indeed transcribed and do not contain additional protein domains in addition to the homeodomain. All previously described planarian HOX gene orthologs were contained in the *S. mediterranea* HOX clones (often >90 % amino acid identity over the full-length protein between different planarian species).

In order to determine whether planarian HOX genes exist in a physical genomic cluster, we summed the size of all genomic contigs containing the 13 HOX genes and also summed the number of predicted genes on each contig, which ranged in size from 13.5 to 327.6 kilobases [54]. In total, the *S. mediterranea* HOX genes span at least 1.75 megabases with at least 50 non-HOX genes in between. It should be noted that in only one instance was more than a single HOX gene found per single genomic contig, suggesting either the largest HOX cluster ever described or, more likely, a fragmented HOX cluster (i.e., “atomized”, see below). In our search through all homeodomain-containing genes, we also found orthologs to the segmentation genes *engrailed, distelless,* and *even*-*skipped*, which were included as positive controls for phylogenetic assignment of *S. mediterranea* HOX genes as well as outgroups. These data strongly suggested that *S. mediterranea* HOX genes are not in a cluster.

### Phylogenetic analysis shows that planarian HOX genes represent all major axial categories

**Fig. 1:**
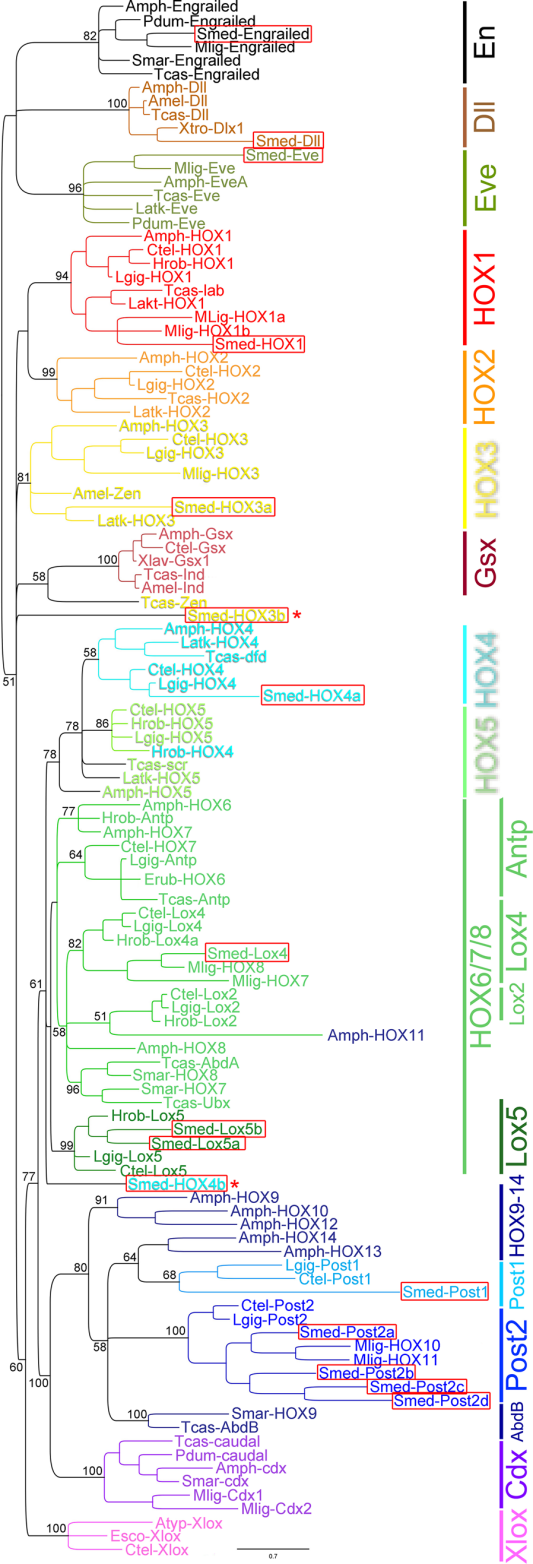
HOX complement and phylogenetic assignments in *S. mediterranea*. A Bayesian phylogeny of select animal HOX genes as well as caudal (Cdx), even-skipped (Evx), engrailed (En), Gsx/ind, and Xlox is shown. Genes from *S. mediterranea* are boxed in *red*. Two genes from *S. mediterranea* could not be placed with confidence: HOX3b and HOX4b, which are marked with red asterisks. Of note, *S. mediterranea* has 4 Post-2 paralogs and no Cdx, Gsx, or Xlox genes. The tree used the engrailed sub-tree as an outgroup, and only the posterior probabilities are shown for important nodes (maximum likelihood tree shown in Additional file 2: Fig. S3). Species and sequences used in the phylogeny are listed in Additional file 1: Table S1. Briefly: Smed = *Schmidtea mediterranea*; Ctel = *Capitella teleta* (polychaete annelid); Amph = Amphioxus (cephalocordate; mix of *Branchiostoma* species *floridae* and *lanceolatum*); Tcas = *Tribolium castaneum* (flour beetle); Latk = *Lithobius atkinsoni* (centipede); Mlig = *Macrostomum lignano* (basal flatworm); Smar = *Strigamia maritima* (centipede); Pdum = *Platynereis dumerilii* (polychaete annelid); Erub = *Ethmostigmus rubripes* (giant centipede); Hrob = *Helobdella robusta* (leech, annelid); Lgig = *Lottia gigantea* (owl limpet mollusk); Amel = *Apis mellifera* (honeybee); Xtro = *Xenopus tropicalis* (frog); Atyp = *Archaster typicus* (starfish, hemichordate); Esco = *Euprymna scolopes* (bobtail squid mollusk)

As HOX gene orthology can be difficult to assign due to a single short stretch of protein homology (60 amino acids), HOX genes are typically divided into 3 major axial categories: anterior (HOX1-5), central (HOX6-8), and posterior (HOX9-14). In order to assign identity, the *S. mediterranea* HOX genes were aligned with arthropod (flour beetle, honeybee, and centipede), annelid (leech and polychaetes; Lophotrochozoan), mollusk (owl limpet *Lottia gigantea*; bobtail squid *Euprymna scolopes*; Lophotrochozoans), basal flatworm (*Macrostomum lignano*), and cordate (amphioxus, starfish, and frog) HOX gene sequences. These species were chosen due to their relatively full HOX gene complements, diverse phylogenetic positions, and reasonable branch lengths (i.e., slow evolving). Finally, alignments were subjected to phylogenetic analysis (Fig. 1; Additional file 1: S3; see “Methods” section) [56, 57].

In general, HOX genes 1–5 can typically be resolved as orthologs from invertebrates to vertebrates. An exception can be the insect *zerknult* (*zen*), which is thought to be a HOX3 homolog [58]. In our phylogenetic analysis, we find *S. mediterranea* orthologs of HOX1, HOX3, and HOX4. We find no support for HOX2 nor HOX5 orthologs, despite orthologs present in other Lophotrochozoans (Fig. 1). Thus, we propose that HOX2 and HOX5 have been lost in triclads (perhaps all flatworms as none of these orthologs were found in *Macrostomum* either).

Two *S. mediterranea* HOX genes could not be resolved (red asterisks; HOX3b, HOX4b). Top reciprocal BLAST hits to the putative HOX3b gene included *zen/HOX3*, and the paraHOX genes *Gsx* and *pancreas/duodenum homeobox protein 1* (*pdx1;* vertebrate *Xlox*). Previously, this Smed-HOX3b gene was described as the paraHOX gene Xlox in a closely related planarian species, *Schmidtea polychroa* [31]. In our analysis, we found little phylogenetic support for this gene being either an *Xlox* or *Gsx* ortholog. Therefore, we propose that this is a degenerate HOX3 paralog and have thus named it *Smed*-*HOX3b* (see “Discussion” section). All the top reciprocal BLAST hits for the putative HOX4b gene were HOX4. Previously, this ortholog was also annotated as a HOX4 homolog in the planarian *Dugesia japonica* [34].

HOX genes 6–8 in deuterostomes correspond roughly to ftz, Ubx, Antp, and AbdA in Ecdysozoa, yet orthology is murky [6, 18, 59, 60]. In Spiralia, these genes are named as Lox2, Lox4, Antp, and Lox5 to reflect Lophotrochozoan HOX differences [61, 62]. Here, we observe that *S. mediterranea* sequences not only cluster with other Lophotrochozoan HOX genes, but *S. mediterranea* has a Lox4 and two Lox5 paralogs and appears to have lost Lox2 and Antp (Fig. 1). As expected for the central HOX genes, the posterior probabilities were not ideal in our analysis, yet the Lox4 and Lox5 groups were well supported, and only one clear error of amphioxus HOX11 was misplaced (Fig. 1).

The posterior HOX genes correspond to a single ecdysozoan gene AbdB, the Spiralian genes Post-1 and Post-2, and the deuterostome genes HOX9-14 [61]. The *S. mediterranea* genes group with the Lophotrochozoan Post genes, with a single Post-1 gene and 4 Post-2 paralogs (named Post-2a-d). Finally, the paraHOX genes Xlox, Cdx, and Gsx could not be found in *S. mediterranea* as has been previously reported in other parasitic flatworms and triclads [3, 18, 31, 63]. In total, almost all *S. mediterranea* HOX genes could be assigned to specific HOX groups, and representatives of all major axial categories were found.

### Multiple methods confirm HOX gene expression in adult *S. mediterranea*: 5 have axially restricted expression; 2 have radial expression; and 6 have tissue-specific expression

**Fig. 2:**
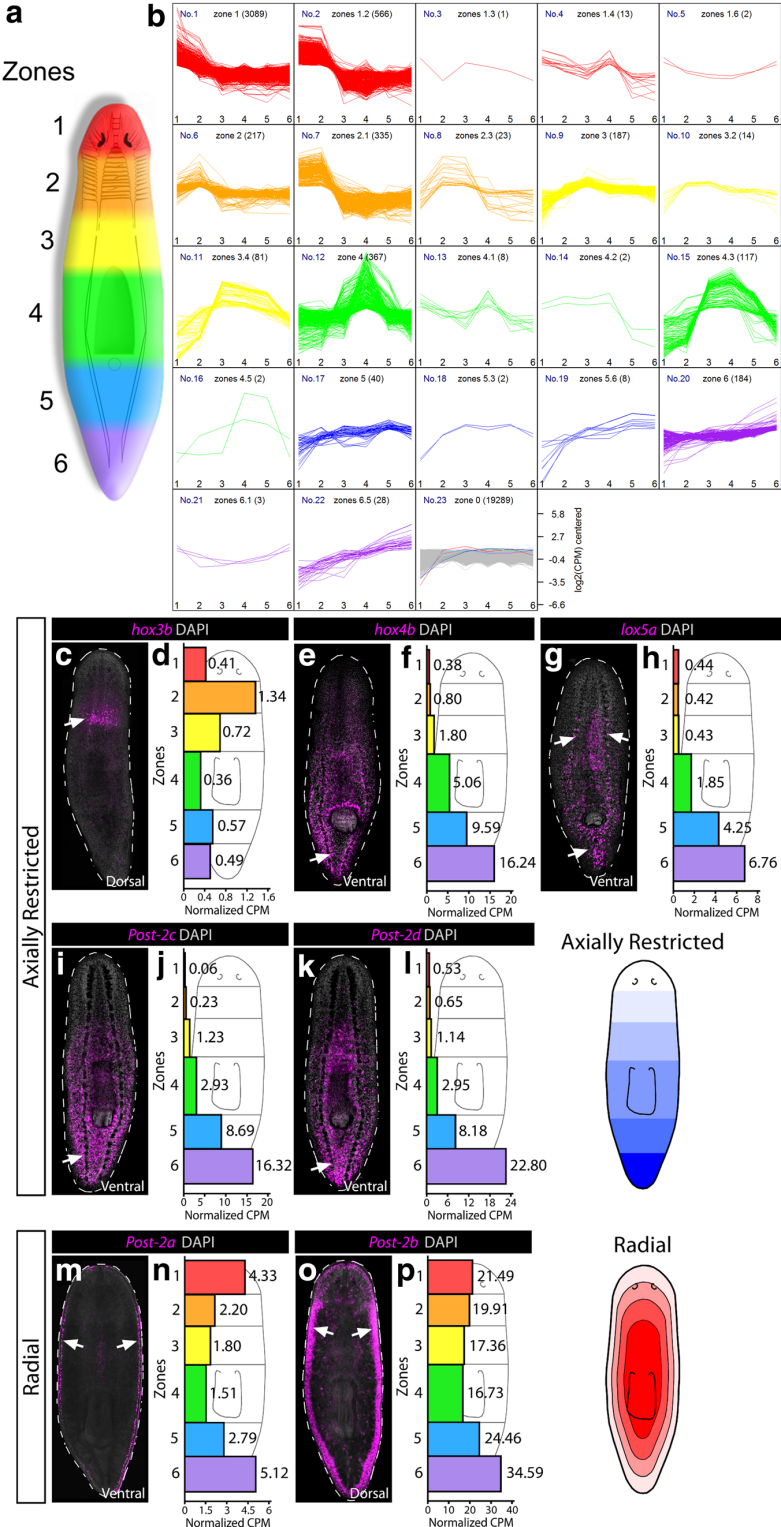
RNAseq and WISH reveal axially restricted HOX genes. **a** A cartoon schematic of 6 axial zones of tissue subjected to RNAseq is shown at the *left*. **b** Following transcript normalization across all zones, transcripts were binned according to having specific expression in particular zones. Twenty-two binning categories were distinguished, of which 16 contain specificity to more than one zone. For example, a specificity of 1.2 would signify that there was specificity to zones 1 and 2, with higher expression in zone 1 (whereas 2.1 has the same specificity but higher expression in zone 2). All transcripts and categories are listed in Additional file 3: Table S2. **c**–**p** FISH stains for individual HOX genes are shown in magenta and counterstained with DAPI in *gray*. *Arrows* denote where the strongest area of expression was seen. For each gene, the raw CPM values per zone are given in the histogram to the right of each stain. Two main categories were observed: axial restriction perpendicular to the A–P axis (**c**–**l**) and radial expression inside of the body edge at the D-V boundary (**m**–**p**)

Previous expression studies on HOX genes in planarians suggested that they may have axial restriction in their expression patterns [31–34, 36, 37]. To support any expression data, we first amputated wild-type animals into 6 A–P zones based on anatomical landmarks and performed RNAseq on each zone (see “Methods” section; Fig. 2a). Following collection, sequencing, and normalization (Additional file 2: Figs. S1, S2), these sequencing data were used to confirm HOX WISH expression. In addition, we provide the raw and processed zone data as a resource to the community for gene discovery of axially restricted transcripts, and we find many other transcripts that have some level of axial restriction to a specific zone (5289 transcripts showed axial restriction into 22 distinct categories; Fig. 2a, Additional file 3: Table S2).

Most planarian HOX genes do not have a published expression, and we have also been unable to detect them with previous WISH methods [50, 51]. Thus, we set out to develop a sensitive WISH method in order to detect the remaining genes (see “Methods” section and Additional file 2). Using this method, all 13 HOX genes were detected and fell into 3 categories of expression. First were genes that showed axial restriction (Fig. 2b). Despite having a clear HOX1 ortholog, the most anteriorly restricted was the putative HOX3 homolog (*Smed*-*Hox3b*), which showed specific enrichment to the neck of the animal. Of the remaining 4 axially restricted patterns, 3 have been previously described, which showed posterior gradients as expected [32–34]. We confirmed these expression patterns using our RNAseq dataset.

**Fig. 3:**
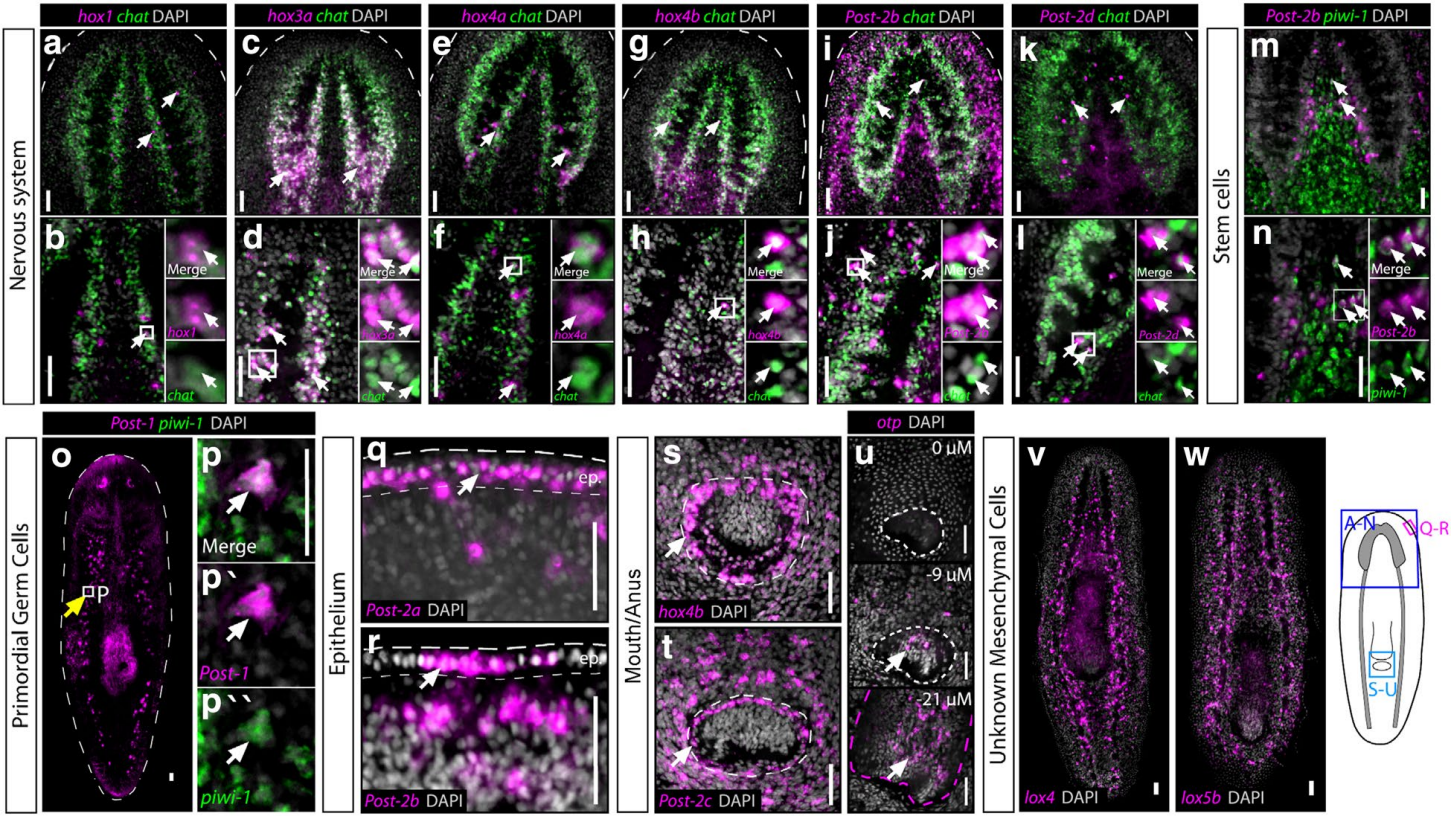
Tissue-specific expression of the HOX genes. For HOX genes that did not show axial restriction by WISH or RNAseq in uninjured animals, all others were detected in specific tissues. Most often, brain and nerve cord expression was detected and confirmed by dFISH with the marker *chat* in *green* to mark cholinergic neurons (**a**–**l**). *Post*-*2b* showed detectable expression in stem cells using the marker *piwi*-*1* (**m**, **n**), and *Post*-*1* was found to be expressed in a small subset of *piwi*-*1* ^+^ cells that are in the anatomical location of primordial germ cells in asexual planarians (**o**, **p**) [71, 72]. The remaining genes were found to be expressed in the epithelium (**q**, **r**), the body opening (**s**, **t**), the pharynx (**u**), as well as unknown mesenchymal cells with broad distribution (**v**, **w**). *Scale bars* = 50 μm

The second category of expression was a radial pattern for the Post-2a and 2b paralogs (Fig. 2m–o). The third and final category of expression displayed little axial restriction and instead showed patterns in specific tissues such as: the brain and nerve cords (Fig. 3a–l); stem cells (Fig. 3m, n); primordial germ cells (Fig. 3o, p); epithelium (Fig. 3q, r), mouth/anus (Fig. 3s, t); and unknown mesenchymal cells (Fig. 3v, w). In total, these data showed that while planarians display some aspects of axially restricted HOX gene expression, many genes have cell-type-specific expression in adult asexual animals.

### Identity of the asexual planarian body opening

Although sexual planarians also have a gonopore body opening, most if not all triclads have a single opening into the gut [28]. It is currently unknown whether this is a mouth or anus, but has been called a “mouth” by convention. HOX expression can help distinguish this, as well as expression of other homeobox-containing genes *caudal* (*Cdx*), *orthopedia* (*otp*), and the T-box gene *brachyury*, which all typically mark the hindgut in other animals [30, 31]. Our analysis suggested that triclads have lost several paraHOX genes, and we could not find a *brachyury* (previously reported in [31]). However, an *otp* was found and the body opening was examined for expression of any HOX genes as well as *otp*. We observed that *Hox4b* and *Post*-*2c* were expressed in the ectodermal body opening, and *otp* was expressed at the end of the pharynx (Fig. 3u; Additional file 2: Fig. S4 C, D). Though not definitive, this is evidence that the pharynx and body opening have posterior character and are a hindgut and anus, respectively (see “Discussion” section).

## Discussion

### HOX gene complement and gene assignments

The data presented here show that planarians have retained a high level of HOX complexity similar to other major phyla of metazoans (between 9 and 14 genes per HOX cluster) [64]. Despite the fact that asexual *S. mediterranea* no longer have embryogenesis, they have retained a 13-gene HOX complement, suggesting that these genes are used in adults in various other processes or during regeneration of their adult tissues [32]. Indeed, 6/13 of *S. mediterranea* HOX genes appear restricted to specific cell types (Fig. 3). In contrast, we present data for 5 other HOX genes, which have clear A–P restriction despite the lack of a HOX cluster (Fig. 2). However, most surprising was the fact that anterior HOX genes showed little axial restriction. We hypothesized that because planarians have a centralized brain and eyes in the anterior, that anterior HOX genes would play a strong role in the regional patterning of these tissues. We could not find any evidence to support this hypothesis, and instead, we now favor a model where anterior HOX genes have been decoupled from adult patterning in *S. mediterranea*. We predict that all the patterning molecules responsible for anterior patterning are captured in the zones 1 and 2 RNAseq data.

Our phylogenetic analyses of *S.mediterranea* HOX genes were in agreement with reciprocal BLAST results for all genes except two. The first, *Smed*-*HOX4b*, was previously annotated in another planarian species as a HOX4 homolog, and all reciprocal BLAST hits to multiple species support a HOX4 identity, yet the gene was incorrectly placed in our phylogenies. Perhaps with more advanced methods or with the inclusion of other flatworm HOX4 sequences, this gene can be resolved into the HOX4 group. *Smed*-*HOX3b* was much more unclear. It was previously annotated as an *Xlox* in a closely related species of planarians [31]. To test this, we added in several Xlox sequences into our dataset and could not find any support for this gene as having an Xlox identity. Similarly, *Smed*-*HOX3b* has high BLAST scores for being a *zen/HOX3* or *Gsx* homolog, and phylogenetic analysis places the gene near the HOX3 and Gsx clusters (Fig. 1). Because Smed-HOX3b could never enter the Gsx group in our phylogeny (100 % support against it), we propose that this gene is a degenerate HOX3, but acknowledge the possibility that it may be a degenerate paraHOX gene Gsx or Xlox.

### Posterior shift of the mouth, anterior shift of the anus, or both?

In planarians, embryogenesis is a highly derived spiral cleavage and potential body openings have not been lineage-traced from embryo to adult. This has made determining the identity of the primary body opening very difficult. In addition, the ectoderm where the opening is located may not necessarily be the entrance into the gut because entrance of food into the gut must go through the pharynx which does not attach to the ectoderm in a traditional fashion. Finally, the pharynx is an anterior/foregut structure in both *C. elegans* and mammals, both of which use a FoxA homolog to specify the tissue [65, 66]. Because *S. mediterranea* also uses *FoxA* to specify the pharynx [67, 68], we previously preferred a model where the pharynx represents the ancestral mouth and entrance into the gut, whereas the ectodermal body opening represents the ancestral anus. In this model, both openings would have shifted axial position to meet in the middle of the animal. To attempt to place an identity of either opening, we examined both for any HOX expression and for expression of the hindgut marker *otp*. We could detect *Hox4b* and *Post*-*2c* in the ectodermal opening (Fig. 3s, t), and surprisingly, we also detected *otp* in the pharynx (Fig. 3u; Additional file 2: S4 C–D). Together, we believe these two patterns of gene expression argue against our initial hypothesis and suggest that the pharynx has hindgut character (although the ectodermal opening is still likely to be an anus). This conclusion also implies that the mouth either was lost or has shifted to the interior of the animal and may exist as the opening where the pharynx enters into the gut. As more genes are examined in these gut openings, it will be interesting to see how the model changes and whether the mouth/anus identity can be clearly determined.

### Future studies on HOX genes in the flatworms: functions and embryogenesis

As more flatworm species are sequenced, it will be possible to determine whether all species of planarians have retained/duplicated the same HOX genes as *S.mediterranea*, as well as determine how some genes were lost in triclads from ancestral flatworms. For example, the basal flatworm *Macrostomum lignano* clearly has *Cdx* homologs, but this paraHOX gene could not be found in any polyclad, cestode, trematode, or triclad. Morphology can differ substantially between species of planarians or other flatworms in general. For example, several members of the planarian genus *Phagocata* have many pharyngeal structures, organized into a segmental-like pattern along the A–P axis (although they still project through the single, ventral body opening) [28]. In addition, parasitic flatworms such as tapeworms have morphological segmentation, but a fairly reduced HOX complement [3, 18]. Through a comparative approach, it will be interesting to examine whether HOX genes play a role in the spatial patterning of these body segments that do not exist in *S. mediterranea*. Finally, it will be important to determine the expression and function of all *S. mediterranea* HOX genes during regeneration, embryogenesis (in sexual species), or simply in the maintenance of A–P spatial identity in adult planarians. A traditional DNA-binding cofactor of HOX genes from the TALE class homeobox family as well as a *teashirt* homolog has strong axial phenotypes following RNAi in planarians [69, 70]. This is suggestive that the HOX genes themselves may play roles in axial patterning in adult planarians. In total, the data here provide the methods and description for future functional studies of flatworm HOX genes and morphological evolution.

## Conclusions

 Here, we perform the first description and expression analysis of the entire HOX complement in a Platyhelminth, the freshwater triclad, *S. mediterranea*. We find that *S. mediterranea* has 13 HOX genes that are not clustered in the genome. Five genes show axial restriction in expression, 2 are expressed radially around the body edge, and the remaining genes have tissue-specific expression. We support the WISH expression data with RNAseq from 6 axial zones of the animal. Interestingly, we detected a posterior HOX gene expressed in the single body opening to the gut. A canonical hindgut marker, *orthopedia*, was also found to have adjacent spatial expression in the pharynx. Together, we propose that the triclad body opening to the gut is an anus and that the mouth has been lost. In total, the improved methods, datasets, and gene descriptions now allow for the functional dissection of HOX gene function in adult planarians as well as during regeneration.

## Additional files

10.1186/s13227-016-0044-8 Sequences and accession numbers used in the phylogenetic analysis in Fig. 1. New *S. med* gene names are given, and homology to any previously described planarian HOX fragments is given as well as references to where they were published. Predicted amino acid sequences are given for any planarian genes as well as the primary homeobox-containing ORF from *Macrostomum lignano* transcripts. All GenBank accession numbers for genes used in the phylogenies in Fig. 1 and S3 are listed.
10.1186/s13227-016-0044-8 Contains supplemental Figs. 1–4 as well as a detailed WISH protocol.
10.1186/s13227-016-0044-8 Zone assignment of transcripts from normalized RNAseq data. The raw data for the zone plots of transcript expression in Fig. 2A are given here. Only genes with normalized CPM > 0.5 are shown.

## Abbreviations

*S. mediterranea*: *Schmidtea mediterranea*
WISH: whole-mount in situ hybridization
FISH: fluorescent in situ hybridization
dFISH: double fluorescent in situ hybridization
RNAi: RNA interference
RNAseq: mRNA deep sequencing
otp: orthopedia
Cdx: caudal
Gsx: GS homeobox
zen: zerknult
A–P: anterior–posterior
CPM: counts per million reads
