# Supplementary material for: HOX gene complement and expression in the planarian Schmidtea mediterranea
Source: EvoDevo. 2016 Mar 30;7:7. doi: 10.1186/s13227-016-0044-8 (PMC4815179; doi:10.1186/s13227-016-0044-8)
Supplement: Supplementary file 2 — 10.1186/s13227-016-0044-8 Contains supplemental Figs. 1–4 as well as a detailed WISH protocol. [file 13227_2016_44_MOESM2_ESM.doc]

**Supplemental Figure legends. Detailed FastBlue WISH protocol**


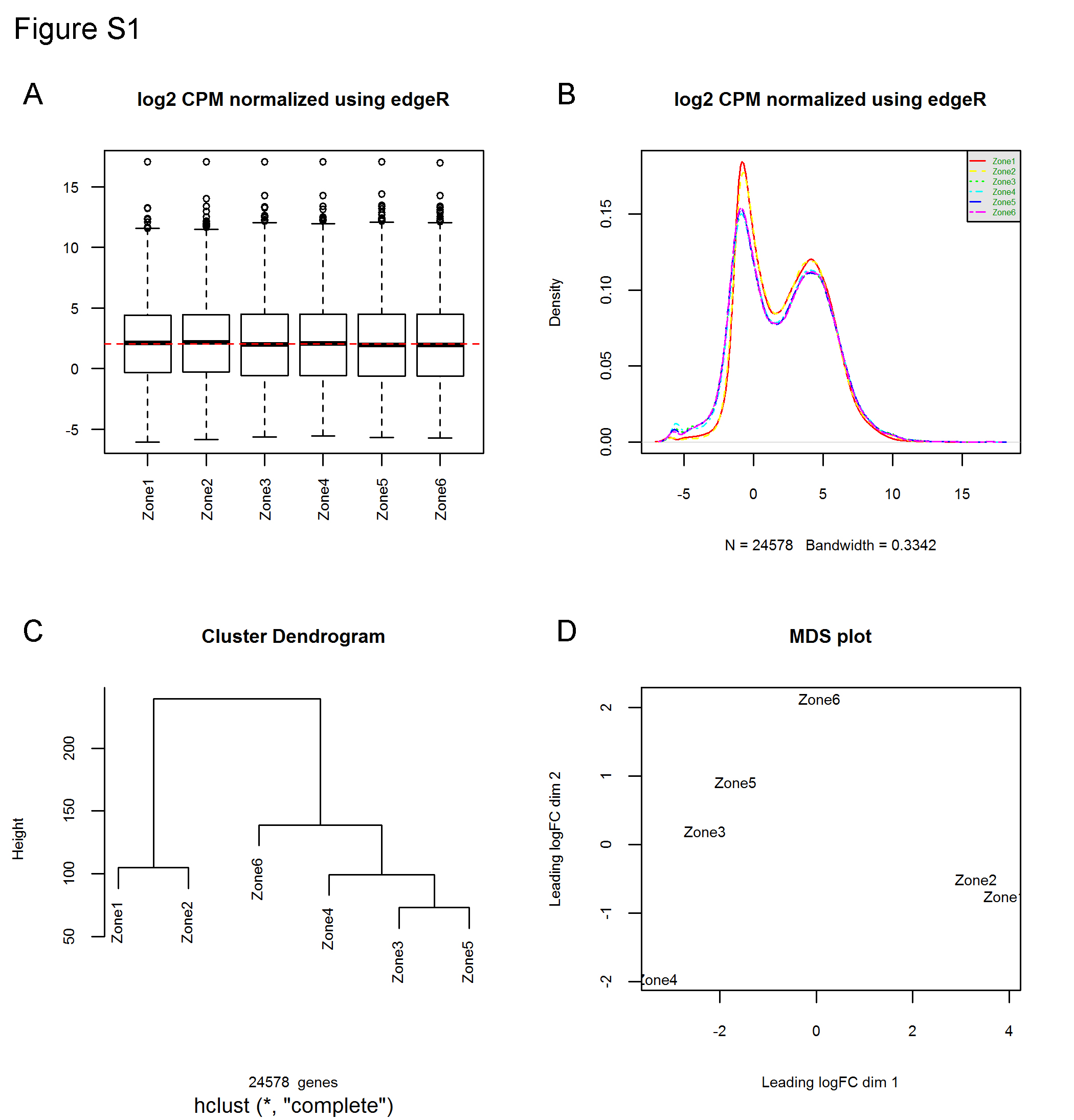
**Figure S1:**

**Normalization of RNAseq Zone data.**

(A) Box-plot, (B) smoothing density-plot, (C) hierarchical clustering, and (D) multidimensional scaling (MDS) were used to show the zone relations after normalizing. The top plots show that the sample distributions for each zone are approximately identical. This means that the normalization has worked. In the following, CPM represents the normalized CPM.


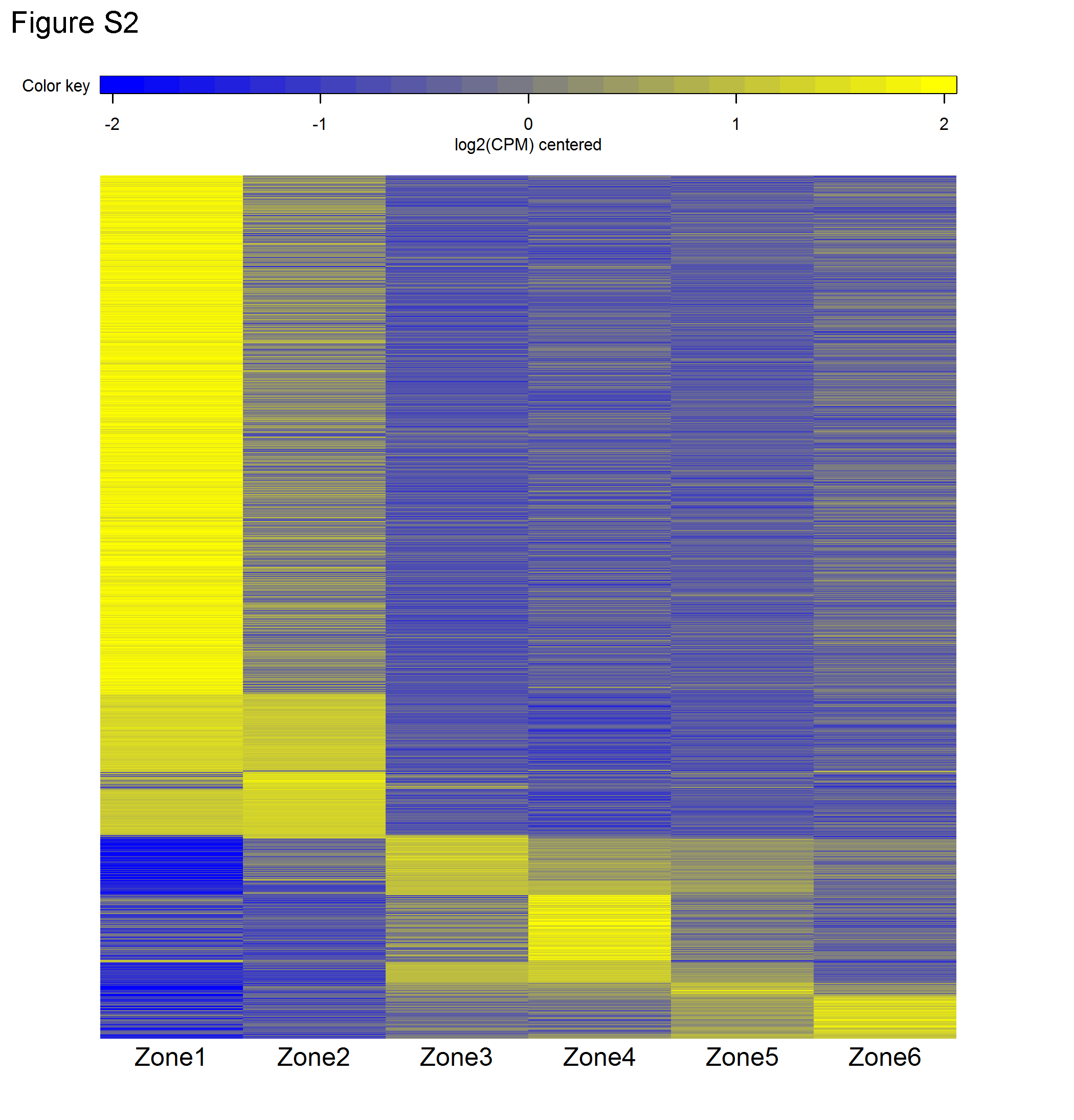
**Figure S2:**

**Heatmap visualization of zone data and distribution.**

Heatmap of log2 normalized CPM for all transcripts except for those not specific to any zone. The rows of heatmap were sorted by the specific zones from 1 to 6. Using edgeR, the specific zones were determined using p-value scores under the significant level 0.05.


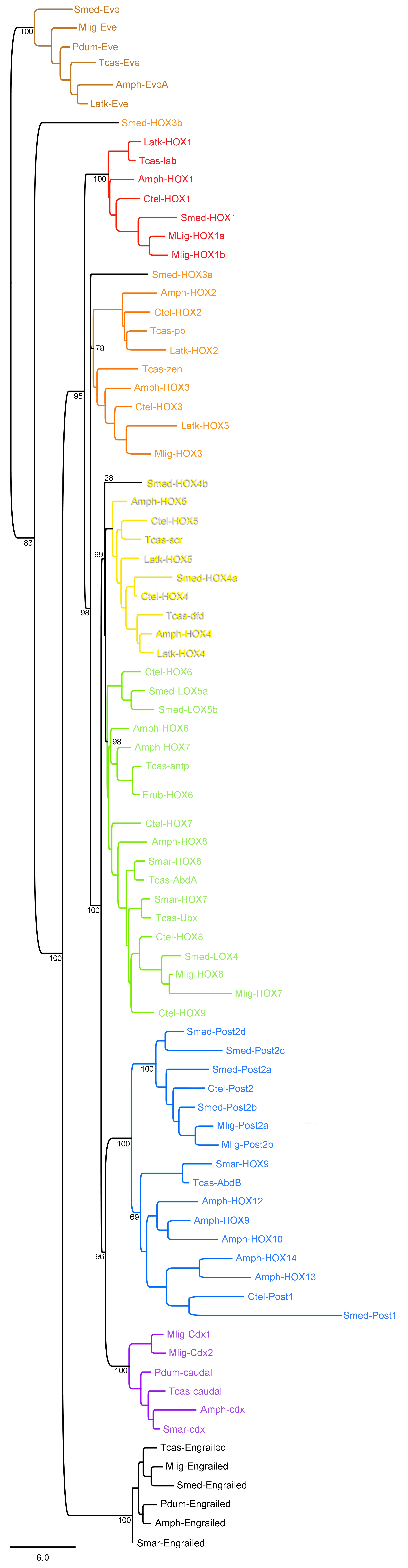


Figure S3

**Figure S3:**

**Maximum Likelihood HOX phylogeny.**

A Maximum Likelihood phylogeny of select animal HOX genes as well as, Caudal, Even-skipped, and Engrailed, is shown. Genes are the same as in Fig. 1 and all *S. med* genes were named based on their top reciprocal BLAST hit. Two genes from *S. med*. could not be placed with confidence: HOX3b and HOX4b. The tree used the Even-skipped sub-tree as an outgroup, and only the bootstrap consensus values are shown for important nodes. Species and sequences used in the phylogeny are listed in Table S1 and Fig. 1 legend.


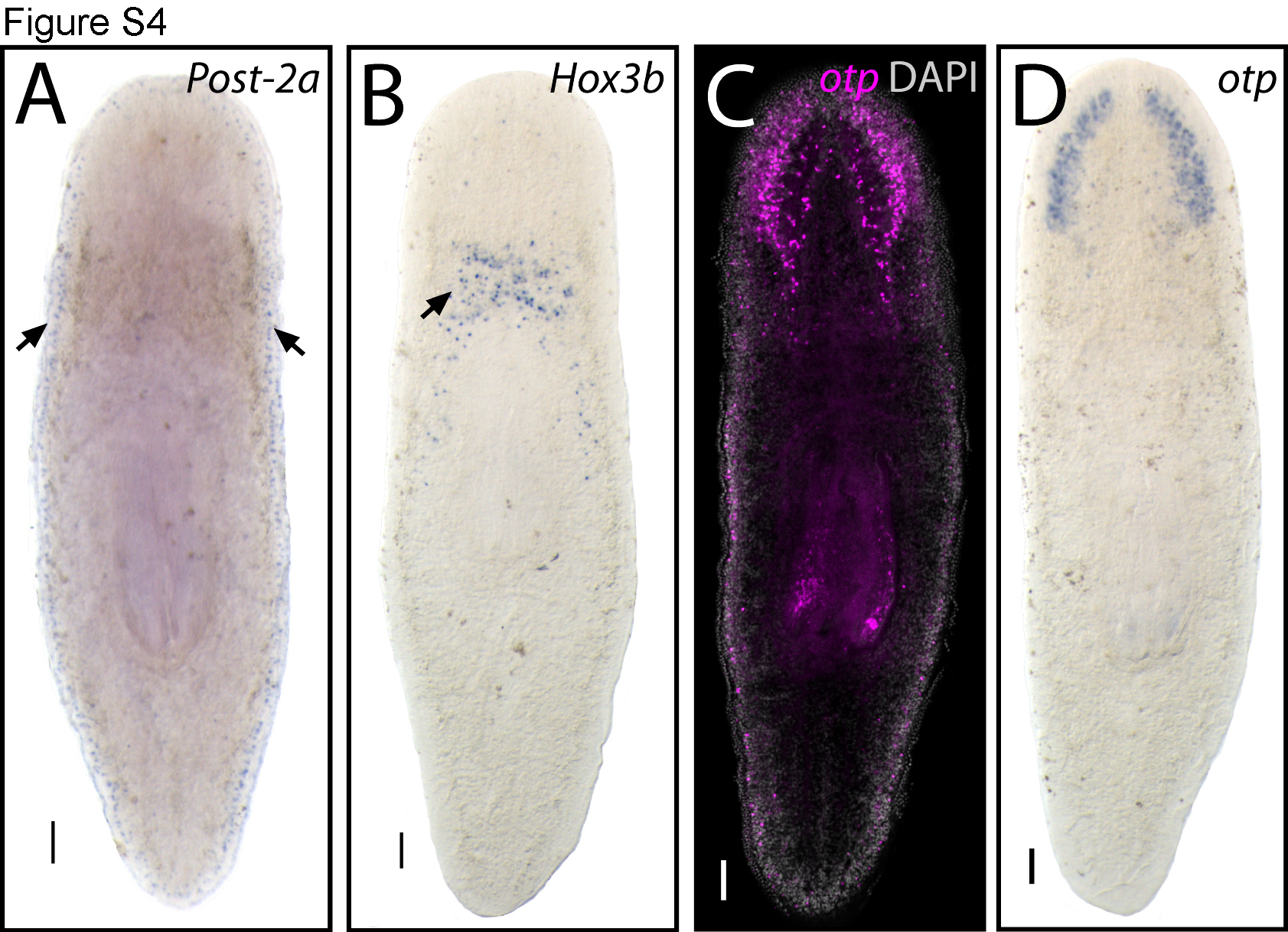


**Figure S4: Colormetric detection of HOX genes and *otp*.**

The HOX genes in Fig. 2 could be detected colormetrically using previous methods and have been reported elsewhere with the exceptions of Hox3b and Post-2c. (A-B) Examples of HOX genes that can be detected colormetrically. (C-D) *otp* expression with Fastblue in magenta vs. colormetric to illustrate the increased sensitivity of the improved method as well as the entire whole-mount expression pattern of *otp*.

**FastBlue WISH protocol, notes, and troubleshooting tips**

**REAGENTS**

**1. Riboprobe Synthesis**

- T7, T3, or SP6 RNA Polymerase (Fermentas; 20U/µL; EP0111)
- 5x Transcription Buffer (Fermentas; comes with RNA polymerases)
- RNaseOUT (Invitrogen; 40U/ µL; 10777-019; Recombinant Ribonuclease Inhibitor)
- DIG-UTP Riboprobe Labeling Mix (Roche; 10x; 11277073910)
- 2x Carbonate Buffer (120mM Na_2_CO_3_; 80mM NaHCO_3_; pH to 10.2, diluted in RNAse-Free H_2_O, aliquot and store at -20^o^C)
- Stop Solution (200mM NaAc; pH to 6.0 with Acetic Acid, diluted in RNAse-Free H_2_O, aliquot and store at -20^o^C)
- RNAse Free water (Invitrogen; AM9937)
- 100% Ethanol, 70% Ethanol, ICE COLD!
- Glycogen (Roche; 20mg/ml; 10901393001)

***in situ* Protocol**

**General Buffers**

- 20x SSC Stock solution (Sigma; S6639)
- 10x PBS Stock solution (Roche; 11666789001)
- PBSTx
  - 0.5% (v/v) Triton X-100 (Bio Basic; TB0198)
  - 1x PBS (from a 10x Stock)
  - Diluted in H_2_O

**Day 1** (kill animals, remove mucus, fix, reduce/permeabilize, dehydrate, and bleach)

- 5% NAC Solution
  - 5% (w/v) NAC (N-acetyl-L-cysteine; Sigma; A7250)
  - Dissolved in 1x PBS
- 4% Fixative Solution (See Note 1)
  - 4% (v/v) Formaldehyde (technically formalin) Solution (Sigma; F8775)
  - Diluted in PBSTx from the 37% stock solution
- Reduction Solution (See Note 2)
  - 50mM DTT (Bio Basic; DB0058; DL-Dithiothreitol)
  - 1% (v/v) NP-40 (Bio Basic; NDB0385) from a 10% stock in H_2_O
  - 0.5% (w/v) SDS (Bio Basic; SB0485) from a 10% stock
  - Bring to volume with 1x PBS
- 50% Methanol Solution(1:1 Methanol:PBSTx)
- 100% Methanol
- Bleaching Solution (By King and Newmark, 2013) (**Warning:** See Note 3)
  - 1.2% H_2_O_2_
  - 0.5x SSC (From 20x SSC Stock)
  - 5% (v/v) Formamide (Bio Basic; FB0211)
  - Bring to volume with H_2_O

**Day 2** (rehydrate, hybridize)

- Hybridization Solution
  - 50-55% (v/v) De-ionized Formamide
  - 5-10% (w/v) Dextran Sulfate (Bioshop; DEX001.50)
  - 5x SSC (From 20x SSC Stock)
  - 1x Denhardt’s Solution (Sigma; D2532)
  - 100ug/mL Heparin from porcine intestine(Sigma; H3149)
  - 1mg/mL yeast torula RNA (Sigma; R6625)
  - 1% (v/v) Tween-20 (BioBasic; TB0560; from a 10% stock)
  - Bring to volume with RNAse-Free H_2_O (Invitrogen; AM9937)
- Pre-Hybridization Solution (Same as hybridization but omit Dextran Sulfate and top up with RNAse-Free water instead)
- 1:1 Pre-Hybridization Solution:PBSTx
- Riboprobe Mix (Hybridization Solution with desired concentration of riboprobe added) (See Note 4)

**Day 3** (washing and antibody incubation)

- SSCTx (2x, 0.2x, 0.05x; all with 0.1% (v/v) Triton X-100, made from 20x SSC stock diluted in milliQ H_2_O)
- Horse Serum (Sigma; H1138; Heat Inactivated and sterile-filtered)
- Western Blocking Reagent (Roche; 10%; 11921673001)
- Block Solution (5% Horse Serum and 0.5% Western Blocking Reagent from 10% Stock. Diluted in milliQ H_2_O)
- Antibody Solution (Block solution with desired antibody (or antibodies) added) (See Note 5)
- MABT
  - 100mM Maleic Acid (Sigma; M0375)
  - 150mM NaCl
  - 0.1% (v/v) Tween-20
  - Bring to volume with milliQ H_2_O and pH to 7.5 with NaOH, autoclave

**Day 4** (antibody washes and development)

- MABT

**Detection/Development**

**NBT/BCIP Colorimetric Development**

- Nitro Blue Tetrazolium (Roche; 11383213001; NBT)
- 5-Bromo-4-chloro-3-indolyl phosphate (Roche; 11383221001; BCIP)
- AP Buffer
  - 100mM Tris, pH 9.5
  - 100mM NaCl
  - 50mM MgCl_2_
  - 0.1% Tween-20
  - Bring to volume with water or a 10% PVA stock solution in milliQ H_2_O (Sigma; P8136; polyvinylalcohol) (See Note 6)
- Development Buffer (AP-Buffer with the addition of 4 µL/mL each of both NBT and BCIP)
- 80% Glycerol Solution (80% Glycerol, 10mM Tris pH 7.5, 1mM EDTA)

**Tyramide Signal Amplification Fluorescent Development**

- Fluorescein-NHS ester (Pierce; 46100) (**Warning:** See Note 7)
- Rhodamine-NHS ester (Pierce; 46406) (**Warning:** See Note 7)
- AMCA-NHS ester (Pierce; 33005) (**Warning:** See Note 7)
- Cy3-NHS ester (Amersham (GE); PA13101) (**Warning:** See Note 7)
- Dimethyl Formamide (Sigma; 227056)
- Triethylamine (Sigma; T0866)
- Tyramide (Sigma; T2879)
- Tyramide Development Buffer (PBSTx with desired tyramide dilution)
  - FITC-Tyramide is used at ~1:2000
  - Rhodamine-Tyramide is used at ~1:5000
  - AMCA-Tyramide is used at ~1:1000
  - Cy3-Tyramide is used at ~1:500
- H_2_O_2_ 30% stock solution
- 80% Glycerol Solution (80% Glycerol, 10mM Tris pH 7.5, 1mM EDTA)

**Fast-Blue Colourimetric/Fluorescent Development**

- Fast Blue BB Salt Hemi (Zinc Chloride) Salt (Sigma; F3378; Fast Blue BB)
- Fast Blue BB Stock (100mg/mL Fast Blue BB Salt in dimethyl formamide; store at -20˚C in small aliquots)
- Napthol-AS-MX-Phosphate Disodium Salt (Sigma; N5000; NAMP)
- NAMP Stock (100mg/mL NAMP in DMSO; store at -20˚C in small aliquots)
- SB8.2
  - 100mM Tris, pH 8.5
  - 100mM NaCl
  - 50mM MgCl2
  - 0.1% Tween-20
  - Bring to volume with water or a 10% PVA stock solution in milliQ H_2_O (Sigma; P8136; polyvinylalcohol) (See Note 8)
- Fast Blue Staining Solution
  - SB8.2 Buffer, split into two equal aliquots
    - To first half add Fast Blue BB stock to a final concentration of 0.75 or 0.5 mg/mL
    - To the other half add NAMP stock to a final concentration of 0.75 or 0.5 mg/mL
    - Add the NAMP/SB8.2 mixture drop wise to the Fast Blue BB/SB8.2 mixture while vortexing slowly to minimize bubble formation.
- TNT Buffer (100mM Tris pH 7.5, 150mM NaCl, 0.1% Tween-20, bring to volume with milliQ H_2_O)
- 80% Glycerol Solution (80% Glycerol, 10mM Tris pH 7.5, 1mM EDTA)

**Antibodies**

- Anti-DIG-AP Antibody (Roche; 11093274910)
- Anti-DIG-POD Antibody (Roche; 11207733910)
- Anti-Fluorescein-POD Antibody (Roche; 11426346910)
- Anti-DNP-HRP or AP (PerkinElmer DNP kits; NEL746A001KT and **NEL747A001KT)**

**PROCEDURES**

**Probe Synthesis**

1. Prepare synthesis reaction as follows on ice in this order in a 500μl RNAse-Free microfuge tube:
   - RNAse-Free H_2_O to 25 µL
   - 5 µL 5x Transcription Buffer (Fermentas)
   - ~10 µL DNA Template (400ng)
   - 1 µL RNaseOut
   - 2.5 µL Dig Riboprobe Labeling Mix
   - 2 µL T7, T3 or SP6 RNA Polymerase (20 U/µL)
2. Incubate reaction at 37˚C for 2 hours
3. Destroy DNA template by adding 2 µL RNAse-free DNAse I (1 U/µL) and incubate reaction at 37˚C for 15 minutes.
4. *Optional: Save 2 µL of the reaction for gel analysis.*
5. Hydrolyze probe by adding 20 µL RNAse-Free H_2_O and 40 µL 2x Carbonate Buffer and incubating at 65˚C for 2.5 minutes.
6. Neutralize probe by adding 80 µL of Stop Solution, mix and put probe on ice.
7. Add 2 μl of glycogen and 220 μl of ice cold 100% ethanol. Mix well!
8. Centrifuge at > 13,000g for 30 minutes at 4^o^C to pellet.
9. Rinse pellets gently with 500μl ice cold 70% Ethanol. Invert once.
10. Re-pellet by centrifugation at > 13,000g for 10 minutes.
11. Decant and air dry carefully (~ 5 minutes). Do not over dry.
12. *Optional: If running a diagnostic gel, suspend the above pellet in 20 µL RNAse-Free H_2_O first, save 2 µL of the hydrolyzed probe for analysis and then add the remaining 80 µL of Pre-Hybridization Solution. Once the probe is resuspended in prehyb, a gel cannot be run due to the torula RNA.*
13. Resuspend probe with 100 µL of Pre-Hybridization Solution or deionized formamide (see Materials ‘*in situ* Protocol’ Day 2) by vortexing and store at -80^o^C.

***in situ* Protocol (Important: See Note 9)**

**Day 1 (kill animals, remove mucus, fix, reduce/permeabilize, dehydrate, and bleach)**

1. Transfer 7-day starved asexual planarians between 2-5 mm to a 1.5 mL microfuge tube (for processing up to 30 animals) or 15 mL Falcon tube (for processing up to 500 worms). (See Note 10)
2. Remove planarian water and replace with 5% NAC Solution and incubate for 5-10 minutes at room temperature (RT). (See Note 11)
3. NAC Solution is replaced with 4% Fixative Solution for 15-20 minutes at RT. (See Note 12)
4. Fixative Solution is removed and worms are rinsed 2x with PBSTx for 5 minutes at RT.
5. PBSTx is replaced with Reduction Solution for 5 minutes at 37˚C. Do not nutate worms at this step as they are fragile. (See Note 11)
6. Remove Reduction Solution and rinse worms 1x with PBSTx for 5 minutes at RT.
7. PBSTx is replaced with 50% Methanol Solution for 5-10 minutes at RT.
8. 50% Methanol Solution is replaced with 100% Methanol for 5-10 minutes at RT.
9. Worms are then stored at -20˚C for at least 1 hour. (See Note 13)
10. Bring worms back to RT and replace 100% Methanol with 50% Methanol Solution for 5-10 minutes.
11. 50% Methanol Solution is replaced with PBSTx for 5 minutes.
12. PBSTx was replaced with Bleaching Solution under direct light for 2 hours. (See Note 14)

**Day 2 (hybridization)**

1. Bleaching Solution is removed and worms are rinsed 2x with PBSTx for 5 minutes each wash.
2. Wash worms in 1:1 PBSTx:Pre-Hybridization Solution for 10 minutes at RT.
3. 1:1 PBSTx:Pre-Hybridization Solution is replaced with Pre-Hybridization Solution for 2 hours rotating in a Hybridizer Oven at 56˚C.
4. Make Riboprobe Mix and incubate at 80˚C for 5 minutes and then place at 56˚C until ready to use.
5. Pre-Hybridization Solution is replaced with Riboprobe Mix and incubated >16 hours at 56˚C without rotation. (See Note 15)

**Day 3 (washing and antibody incubation)**

**NOTE: All wash solutions should be brought up to and kept at 56˚C prior to use.**

1. Riboprobe Mix is carefully removed and replaced with 2x SSCTx. (See Note 16)
2. Worms are rinsed in a 56^o^C Hybridizer Oven by the following steps:
   - Rinse 2x with 2x SSCTx for 30 minutes each wash. (See Note 17)
   - Rinse 2x with 0.2x SSCTx for 30 minutes each wash.
   - Rinse 2x with 0.05x SSCTx for 30 minutes each wash.
3. Return worms to RT and wash with MABT 2x for 10 minutes each wash. (See Note 18)
4. Transfer worms to a 24-well plastic plate and replace MABT Solution with Block Solution for 2 hours at RT.
5. Replace Block Solution with Antibody Solution and incubate for overnight at RT.

**Day 4 (antibody washes and development)**

1. Antibody Solution is replaced twice quickly with MABT. (See Note 19)
2. Worms are then washed an additional 6x with MABT over 2 hours.
3. Develop worms as per selected development procedure.

**Development Procedures**

**NBT/BCIP Colorimetric Development**

1. Replace MABT Solution with AP Buffer for 10 minutes at RT.
2. AP Buffer is replaced with Development Buffer and worms are placed in the dark.
3. Develop until optimal signal to noise ratio is attained. (See Note 20)
4. Stop the development by replacing Development Buffer with PBSTx 2x 5 minutes.
5. Post-fix worms by replacing PBSTx with 4% Fixative Solution for 10 minutes at RT.
6. 4% Fixative Solution is replaced with PBSTx.
7. PBSTx is replaced with 100% Ethanol for ~20 minutes at RT to remove non-specific background staining. (See Note 21)
8. 100% Ethanol is replaced with PBSTx.
9. PBSTx is replaced by 80% Glycerol Solution and stored at 4˚C to clear worms.
10. Cleared worms (no longer floating) are transferred to a slide and mounted under a #1-weight coverslip.
11. Mounted slides are stored at 4˚C until required for imaging and should be stable for > 6 months.

**Tyramide Signal Amplification (TSA) Fluorescent Development**

**Tyramide Conjugate Preparation (Work in a Fume Hood):**

1. Generate a 10mg/mL stock of desired NHS-ester in dimethyl formamide (DMF).
2. Generate DMF-TEA Solution (1mL DMF to 10uL triethylamine).
3. Generate Tyramide Solution (10mg tyramide-HCl to 1mL DMF-TEA)
4. Create Reaction Solution (4:1.37 NHS Ester Stock:Tyramide Solution)
   - Mix and incubate at RT in the dark for 2 hours.
   - Then add 0.115mL EtOH per mg of NHS-ester used and store at -20^o^C in the dark. This Solution has been found to be stable indefinitely.

**Development:**

1. MABT is replaced with PBSTx for 30 minutes at RT.
2. Replace PBSTx with Tyramide Development Solution and incubate at RT for 30 minutes.
3. Initiate development by adding H_2_O_2_ to a final concentration of 0.002-0.015% and place worms in the dark. (See Note 22)
4. Develop the worms for 45 minutes. (See Note 23)
5. Replace Tyramide Development Solution with PBSTx.
6. Wash worms with PBSTx several times. (See Note 24)
7. PBSTx is replaced by 80% Glycerol Solution and stored at 4˚C to clear worms.
8. Cleared worms (no longer floating) are transferred to a slide and mounted under a #1-weight coverslip.
9. Mounted slides are stored at 4˚C until required for imaging.

**Fast-Blue Colorimetric/Fluorescent Development for dFISH 3-day protocol**

**Note:** *This protocol is adapted from Lauter et al., 2011. It involves the use of an alkaline phosphatase driven reaction to produce a precipitate that can be viewed both colorimetrically and fluorescently (in far red). It is an excellent method to make dFISH a more robust method in planarians.*

1. Wash worms 2x with PBSTx for 5 minutes each at RT.
2. Replace PBSTx with SB8.2 for 5 minutes at RT.
3. Initiate development by replacing SB8.2 with Fast Blue Staining Solution and place worms in dark.
4. Monitor development until completed. (See Note 25)
5. Stop development by washing worms 4x 5 minutes with TNT and 2x 5 minutes in PBSTx.
6. Replace PBSTx with 80% Glycerol Solution and store worms at 4˚C to clear.
7. Cleared worms (no longer floating) are transferred to a slide and mounted under a #1-weight coverslip.
8. Mounted slides are stored at 4˚C until required for imaging.

**Notes**

1. Many protocols call for “paraformaldehyde” to be made from scratch. It is possible that the methanol in formalin solution does affect WISH in other systems, however, we cannot detect differences head to head. We stand by our experience that formalin solution is the way to go and can be adapted to any organism, and is vastly safer than working with paraformaldehyde.
2. Similar reagents from Sigma can be used instead.
3. Dilute H_2_O_2_ with H_2_O before adding Formamide, otherwise a violent reaction will result.
4. It is recommended that you try several dilutions of new probes to determine the optimal concentration to minimize noise and maximize signal. Begin at 1:500 dilution.
5. Desired concentration of antibody differs for each antibody used. We use Anti-DIG-AP at 1:4000 and Anti-Fluorescein-HRP at 1:300.
6. Water is used for probes that are found to develop rapidly. PVA is used to enhance development for probes that are weaker in nature.
7. Esters are unstable and highly reactive with water. After opening, dissolve entire contents in anhydrous DMF and use immediately and perform the synthesis protocol.
8. Water is used for probes that are found to develop rapidly. PVA is used to enhance development for probes that are weaker in nature.
9. Intact planarians can be nutated/rocked and regenerating fragments (up to day 2) may be rocked gently unless indicated otherwise. It is also much easier to take solutions off of worms with a glass pipet and bulb.
10. When fixing in in microfuge tubes, use 1 mL of solution volume for these steps. When fixing in a 15 mL tube, use 12 mL of solution volume so that worms do not get beat up.
11. These two solutions are not constants in this protocol, and always require constant balancing. NAC is stronger when it is fresh, and should not be used after it is ~4 months old. In between these times, it steadily loses activity. Therefore, the time of NAC treatment varies from about 4-10 minutes. If worms clump slightly in one fixation, treat with NAC for a little longer next time. If worms lose significant epithelia and are breaking, use less NAC next time. For the reduction solution, the key is how the “neck” region of the worm stains because the neck secretes an additional type of mucus or matrix that resists complete staining, especially in larger worms. Thus, if the neck is lighter staining than the rest of the body, longer reduction times are needed. If the neck stains relatively darker than the body, the worms are over-permeabilized. Most worms in the 2-4mm range do not require much reduction. For 1cm worms, a 15 minute reduction at 37˚C may be required.
12. A key to adding the fix to freshly de-mucused worms is to keep them suspended in the fluid and to avoid settling and clumping.
13. Worms can be stored for 1-2 weeks in methanol until required. For longer storage periods it is advised to store worms in 100% ethanol instead.
14. King and Newmark, 2013.
15. Due to the dextran sulfate in the hyb/riboprobe mix, the solution is very viscous and the animals will often float on the surface. This is ok, but once the riboprobe mix has been added to the animals, the tube should be gently vortexed to thoroughly mix the solution and worms. Take care to not leave worms on the sides of the tubes, out of the solution!
16. Probes can be carefully removed with a p200 pipette and stored at -20^o^C for future use. Probes can often be used 2-3 times, and typically become cleaner (less background) every time. If the probe is not being saved, it is much easier and faster to add 2x SSCTx directly on top of the Riboprobe mix to help the worms sink to the bottom so that subsequent washes can begin.
17. For less-stringent posthybridization washes, you can wash with this sequence: 2x 30 minute washes with 1:1 (Prehybridization Solution:2xSSCTx), 2x 30 minute washes with 2x SSCTx and 2x 30 minute washes with 0.2x SSCTx. See more in “Troubleshooting”.
18. MABT must be used for all AP antibodies as phosphate-containing buffers will kill the enzyme. We have not detected measurable differences for MABT vs. PBSTx vs. TNT for any fluorescent developments, so all 3 buffers can be used if need be.
19. Enzymatic secondary antibodies are not reused.
20. Monitor worms every 5 minutes initially. Continue to develop worms until the pink background starts to turn magenta in colour. This is generally the point at which the background starts to mask signal. Typical stains develop within an hour, the fastest stains develop in about 5 minutes.
21. Monitor this step closely and stop the Ethanol wash when your signal turns blue and your background disappears. This will be only a few minutes for clean stains.
22. It is useful to create a 1/100 dilution of H_2_O_2_ in PBSTx in a separate Eppendorf 1.5mL tube and add it to your wells at 1/100, for a final dilution of 1/10000.
23. To increase development potency spike in H_2_O_2_ to initiate development, and respike again in 20 minutes. At the end of 45 minutes, stop the development.
24. If you are developing a second probe by TSA development, you must first quench your POD or HRP containing antibody to prevent false doubling. To do this incubate worms in 100mM sodium azide for 45 minutes (King and Newmark, 2013).
25. For problematic developments, it can be useful to use an alternative staining buffer (SB8.2-PVA) in which water is replaced by PVA. When using this alternative staining buffer, we have found that shorter incubation periods (~20-30 minutes) produce cleaner results. However, as a result of shorter incubation times, the stain does not always appear colorimetrically.

**METHOD VARIABLES**

Common optimizations that can be performed:

**Probe concentration:**

We are finding that even for difficult probes, lower probe concentrations produces cleaner and clearer stains. This may be the single most important variable to test for any given probe.

**Bleach:**

Traditional bleach in planarians is 6% H_2_O_2_ in Methanol or Ethanol overnight under direct light. The method outlined in this protocol has recently been shown to work better and be faster . It should be noted that it is somewhat unclear whether a bleaching step prior to hybridization is even required, or whether other bleaching methods might work better. Thus, it is possible that the WISH protocol could be optimized for unbleached worms, and then they would be post-bleached to white following development. The treatment with hydrogen peroxide prior to hybridization is also worrisome due to the fact that this oxidizer should be able to hydrolyze the RNAs that we want to detect by WISH. Perhaps the sensitivity of the method can be increased by omitting the bleach step. NaOH based bleaches are used for zebrafish embryos, which could also be applied here.

**Permeabilizations:**

There are many ways to permeabilize tissues. For WISH, the most common is the use of SDS. We have had quite a bit of luck adding 0.1% SDS to our hyb solutions, which almost bleaches the animals white in itself. However, the SDS will precipitate out of the hyb when stored at -20˚C, and can also make animal slightly sticky in posthybridization steps. For these reasons, it is typically omitted, but it is necessary for hybridizing larger animals ( > 1 cm ), so it might be useful in the normal protocol.

**Hyb temp:**

Planarians have a high AT genome (about 70% AT). For this reason, we hybridize at a non-traditional temperature of 56˚C. We have never had success at obtaining clean stains at a more traditional 65˚C. Thus, somewhere between 56˚C and 65˚C is a “too stringent” temperature, which can be used to optimize signal to noise for a given probe. Note that 56˚C is just a starting point and for less signal go up in temperature and for more signal go down.

**Hyb length:**

A variable heavily optimized in other systems but relatively untouched in planarians is the length of hybridization. In other systems, it is not uncommon to replace the hyb solution after 24 hours, and hyb for another 24 hours. Therefore, it is possible this variable could help the detection of very weak probes.

**Hyb recipe:**

There about are as many hyb recipes as there are apple pie recipes. We have obtained very clean and strong stains both from the simple recipe from mouse embryos (50% formamide, 5xSSC, 1%SDS) and our current recipe in this protocol (*i.e.* a very complex recipe). In general, every component can be optimized, but in practice, just about every recipe works to some extent (just like apple pie). One particular variable that has not been optimized in planarians is pH of the hyb.

**Dextran sulfate:**

The most critical component to planarian hyb besides the quality of formamide is the addition of dextran sulfate. This large molecule effectively concentrates probe by sequestering water to stay in solution. In fact, the addition of this to the hyb solutions used in other organisms has had similar effects. It is possible to go even higher in dextran sulfate concentration, or switch over to similarly large hydrophilic molecules such as polyvinyl alcohol (PVA) or polyethylene glycol (PEG). These alternatives have not been tried, but it is possible they may have even more dramatic effects.

**Riboprobes:**

PCR, hydrolysis, and specific regions of the transcript. Riboprobes were conventionally made in the 1990s by using a plasmid template that was restriction digested on the 3’ end so that the polymerase would fall off the template after synthesizing the riboprobe. Testing this against probes made using a PCR-generated template has yielded no significant differences in planarians, and therefore we recommend using PCR templates to make riboprobes. Probe hydrolysis is a tricky issue, but almost always helps staining, regardless of probe length. The only time we do not hydrolyze probes is when we cannot obtain patterns under all other conditions. Making riboprobes to different parts of a given transcript can have vastly different specific activities. Therefore, when staining cannot be obtained, it can be useful to use a subcloned region of a transcript. Even easier is to design a primer with a T7 or T3 site and use the new PCR template for probe making (instead of subcloning).

**Developer (ratios, PVA, O/N at 4°C):**

An important aspect of WISH that is often overlooked is the developing solution and method for colorimetric development. It is not widely known, but NBT/BCIP ratios can be substantially altered to achieve better staining. 5:1 ratios can be used in both directions and empirically tested. Additionally, concentrations can also be increased or decreased to speed up or slow down development. The standard we use is 4 μL of each NBT and BCIP per mL of developing solution. However, we have gone up to 10 μL per mL with good results. PVA is also a critical reagent for developing weaker stains for the same reason dextran sulfate helps hybridization. Although for strong probes that develop within 1 hr, PVA is omitted due to the fact that it restricts diffusion of NBT/BCIP and the result is non-uniform staining. We have not tried PVA in TSA, but it is predicted to also aid development. In addition, dextran sulfate instead of PVA may work better as well. While common in other systems, developing WISH stains in planarians overnight at room temperature has not yielded improved results. However, it has not been tried with very low probe concentrations (*i.e.* < 1/20,000 dilution). The more the background can be minimized, the longer an ISH can be developed. That being said, good results for colorimetric WISH have been obtained by developing overnight at 4˚C in the dark.

**TEA/AA treatments:**

One series of treatments that has worked in other systems and has drastically improved WISH in planarians fixed by Carnoys fixative, is by treating with triethylamine and acetic anhydride. It is thought that these chemicals will acetylate charged molecules in the cells, which will allow less non-specific interactions with riboprobes and lead to lower background. It has yet to be applied in our protocol, but it may help with background issues.

**Proteinase K or collagenase:**

A glaring omission to our protocol that is included in most WISH protocols is a post-fix treatment with proteinases. These proteinases are thought to “free up” the RNAs in the cell that have been fixed to proteins during the fixation procedure. This has been used in planarians to varying success . Proteinase K is the most common and harsh proteinase. A common treatment would be 5 mg/mL of proK in PBSTx for 10 minutes, and then a 10 minute post-fix in fixation solution. Collagenase has also been used in other systems as a less-harsh treatment and could be applied here. In our experience, this treatment is variable, results in more fragile worms, and is not important to achieving robust and sensitive staining.

**TROUBLESHOOTING**

It should be said at the outset that most probes require some form of optimization. Often times, issues can be solved with simple changes in probe concentration or development (see Figure 3). It should also be noted that these troubleshooting tips assume that your probes and solutions work well with other known probes, and thus, are not the cause of the issues in your staining. Below are specific tips on common issues.

**Non-specific or no staining:**

Non-specific staining is defined by no specific signal (dark purple) over the course of about 3-4 hours of development in planarians, after which the worms tend to turn a uniform pink. It is very common to try to obtain a WISH signal for a gene that is expected to have a very specific expression pattern, and instead see non-specific or no staining. Two examples of transcription factors that should have specific expression in the planarian nervous system are shown in Figure. 2. Neither of these probes will develop well with NBT/BCIP or Fast Blue colorimetrically. Part of the reason this protocol was developed is because we knew the genes were expressed by RNA sequencing, but could not detect them with conventional methods. When viewed under confocal microscopy, specific patterns emerge for both of these transcription factors. We believe that using this protocol is the best tip to detect genes that give non-specific/no staining patterns with conventional methods.

It is also possible that non-specific staining can result from: posthyb washes that are too stringent, probes that are too short in length, excessive permeabilization, excessive hybridization temperature, or insufficient antibody concentration. For less stringent posthyb washes, higher salt = less stringent. It is even common in other systems to perform all posthyb washes in prehybridization solution (5xSSC). The important factor here is to empirically determine what washes work for your probe, but try to stick to 6+ washes over 3+ hours, and any combination of buffers from 5xSSC to 0xSSC (*i.e.* no salt). Short probe length isn’t always a solvable problem, but skipping hydrolysis of the probe can dramatically help probes that are < 1KB. 5’ and 3’ RACE to clone the absolute full length gene can help generate longer probes as well. Finally, it is possible in other systems to clone introns and use those as probes to detect foci of transcription in the nucleus (not extensively attempted in planarians, however). This being said, we have achieved fantastic staining with 5KB non-hydrolyzed probes and hydrolyzed 200 base pair probes. The inherent specific activity of a given probe is the biggest factor, and unfortunately, this variable is out of our control.

**High background:**

Unfortunately, the problem of high background is equally as common as non-specific staining, but unlike non-specific staining, it has many causes and many cures. High background is usually defined as when a specific signal can be observed, but probe concentration or development cannot be altered such that the signal to noise ratio will allow the specific signal to be illustrated. The first point here is that sometimes what looks like background is real staining of ubiquitous expression. Second, assuming the observed background is non-specific, then too much hybridization is occurring and stringency must be increased. Two easy ways to increase stringency are to increase hybridization temperature (2 degrees per attempt) and to decrease the salt concentration in posthyb washes (all the way down to 0x SSCTx if need be). If these do not help, more involved methods include skipping the hydrolysis step in riboprobe synthesis (especially true for genes < 1 KB), or subcloning different regions of along the gene’s length to see whether the specific staining can be achieved with less background. Decreasing the anti-DIG-AP antibody concentration to 1/10,000 can also help. Finally, hyb recipes can be altered to help with background. Increasing formamide concentration from 50% yields approximately 1-2 degrees more stringency per percent increase. Conversely, decreasing dextran sulfate is somewhat equivalent to decreasing probe concentration. Addition of more detergents to the hyb (0.1% SDS or 1% CHAPS) can also have dramatic effects on background, although they tend to occur at the expense of animal integrity. Lastly, the nucleic acid blocking component of the hyb can be increased or changed. Some common nucleic acid blocks include sheared salmon sperm DNA, yeast tRNA, or homemade multi-phenol:chloroform extracted yeast total RNA. We choose torula RNA for the reasons that it is cheap, effective, and single stranded RNA to occupy the time of any contaminating RNAses.

**Development easy with NBT/BCIP, impossible with TSA:**

A common problem (and a primary reason for the development of this current protocol) is that a probe develops well with NBT/BCIP, but is virtually impossible to develop with TSA. Clearly the easy fix to this is to develop with Fast Blue, but if two probes need to be developed and are similarly weak, one of them will have to be developed with TSA. One way to accomplish this is to optimize the combinations of: 1) UTP modification; 2) Probe development order; 3) Which tyramide-fluorphore is used. Not all modified UTPs are equal in strength and volatility. Empirically in planarians, we find that from strongest to weakest, UTPs are in this order: DNP>DIG>Fluorescein>Biotin. As mentioned previously, the earlier in the protocol a probe is developed, the more likely it will work. Thus, the TSA probe should be developed first. Finally, lower wavelength fluorophores are typically stronger under confocal microscopy. In general, FITC>Cy3>Rhodamine>Cy5. We have not extensively tested AMCA. If you have 2 probes of similar weakness, you would choose your TSA probe to be developed first. You would use DNP to make the probe, and you would develop with FITC-tyramide. The recommendation for the other probe would be to use DIG and Fast Blue.

There are two additional notes on this. First is that fluorescein and FITC are molecules that are both recognized by the anti-Fluorescein antibodies (HRP and AP). Therefore, once a FITC-tyramide reaction has taken place for a probe, the animal can never again be probed with the anti-Fluorescein antibody. The exception to this statement is the second note. Often times, super weak probes with TSA can be specifically amplified using this antibody cross-talk. For example, a DIG probe can be developed for about 10-20 minutes with FITC tyramide, and then restained with an anti-Fluorescein-HRP antibody, and redeveloped with any color tyramide. This sequential double amplification will often yield very strong staining for probes that do not work with a single TSA.

**Molecular measles:**

A phenomenon called “molecular measles” occurs sometimes in planarians where at first glance it appears that there was real, strong, specific staining. However, the staining is actually located outside of the epithelia of the worm and can be picked off by forceps. We find that this happens most frequently when bacteria and/or fungi are growing in MABT or horse serum. Sterile filtering of MABT and the Blocking Solutions normally takes care of this issue.

**Neck issues:**

The neck region of a planarian is a perfect indicator of whether your fixations and permeabilizations are in balance. This is due to a particularly dense mucus or secretion that exists in the neck, particularly in mid-sized animals (*i.e.* > 6mm). Small worms have little to none of this secretion, permeabilization is not an issue, and the “reduction” step can be skipped entirely. On the other hand, large worms > 1cm in length are very difficult to stain in the neck region, and both NAC and reduction must be increased in time until staining is uniform. 0.1% SDS addition to the hyb also helps on larger animals. In terms of general rules, if the neck staining is weaker than the staining in the body, more permeabilization is needed, and if the neck staining is darker than the body, permeabilization was too long/strong such that real staining in the body has leached out. Thus, when the neck region looks identical in intensity to the rest of the animal, permeabilization is correct.

**Worm integrity issues:**

For people new to staining planarians, their WISH stains will typically work the first time, but the animals will have lost large chunks of epithelia or be broken in half. This is attributed to over-rocking and being sucked into pipet tips. The borosilicate glass pipets and bulbs offer much more control over liquid removal, and when worms enter the pipet, they are not sheared as much. An additional technique to watch is to pipet new solution on the side of the tube/well, instead of directly on the worms. This will also help with integrity. Despite these tips, not enough can be said for how much integrity can be improved with practice!

**REFERENCES**

1. King RS, Newmark PA (2013) In situ hybridization protocol for enhanced detection of gene expression in the planarian Schmidtea mediterranea. BMC Dev Biol 13: 8.

2. Guedelhoefer OCt, Sanchez Alvarado A (2012) Amputation induces stem cell mobilization to sites of injury during planarian regeneration. Development 139: 3510-3520.

3. Pearson BJ, Eisenhoffer GT, Gurley KA, Rink JC, Miller DE, et al. (2009) Formaldehyde-based whole-mount in situ hybridization method for planarians. Dev Dyn 238: 443-450.
